# Increased NOX-dependent ROS production and proportionally enhanced antioxidant response in white adipose tissue of male rats

**DOI:** 10.20945/2359-4292-2024-0136

**Published:** 2024-11-06

**Authors:** Jessica de Freitas Nascimento, Keciany Alves de Oliveira, Paula Alexandre de Freitas, Júlia de Araújo Marques Falci, Renata Prado Vasconcelos, Saulo Chaves Magalhães, Talita Mendes Farias, Maria Isabel Cardoso Alonso-Vale, Adriano Cesar Carneiro Loureiro, Denise Pires de Carvalho, Rodrigo Soares Fortunato, Ariclécio Cunha de Oliveira

**Affiliations:** 1 Universidade Estadual do Ceará Instituto Superior de Ciências Biomédicas Laboratório de Fisiologia Endócrina e Metabolismo Fortaleza CE Brasil Laboratório de Fisiologia Endócrina e Metabolismo, Instituto Superior de Ciências Biomédicas, Universidade Estadual do Ceará, Fortaleza, CE, Brasil; 2 Universidade Estadual do Ceará Programa de Pós-graduação em Nutrição e Saúde Fortaleza CE Brasil Programa de Pós-graduação em Nutrição e Saúde, Universidade Estadual do Ceará, Fortaleza, CE, Brasil; 3 Universidade Federal do Rio de Janeiro Instituto de Biofísica Carlos Chagas Filho Centro de Ciências da Saúde Rio de Janeiro RJ Brasil Centro de Ciências da Saúde, Instituto de Biofísica Carlos Chagas Filho, Universidade Federal do Rio de Janeiro, Rio de Janeiro, RJ, Brasil; 4 Universidade Federal de São Paulo Departamento de Ciências Biológicas Piracicaba SP Brasil Departamento de Ciências Biológicas, Universidade Federal de São Paulo, Piracicaba, SP, Brasil

**Keywords:** Adipose tissue, sexual dimorphism, redox homeostasis, NADPH oxidase

## Abstract

**Objective:**

This study aimed to investigate the redox balance in subcutaneous and retroperitoneal fat pads of male and female Wistar rats.

**Materials and methods:**

The study analyzed the activity and gene expression of the antioxidant enzymes superoxide dismutase, catalase, and glutathione peroxidase, along with the production of NADPH oxidases dependent on H_2_O_2_ and gene expression of NOX1, NOX2, and NOX4.

**Results:**

The retroperitoneal fat pad in males compared with females had greater NOX2 and NOX4 expression, along with higher superoxide dismutase activity. Additionally, their subcutaneous fat pad had greater NOX4 expression and higher intracellular H_2_O_2_ production, together with greater expression and activity of both superoxide dismutase and catalase.

**Conclusion:**

The white adipose tissue of male rats had greater reactive oxygen species (ROS) production compared with that of female rats, but also a proportionally greater antioxidant response. These findings are important for ongoing investigations into how sex differences may be linked to the development of metabolic diseases and the unique susceptibilities of each sex.

## INTRODUCTION

The adipose tissue can be classified according to its origin, color, and function as brown adipose tissue and white adipose tissue (WAT) (1). The WAT has been characterized as a dynamic and central organ in metabolic regulation, synthesizing and secreting hormones, participating in glucose metabolism, and playing a role in important processes, such as inflammation (2). Notably, the WAT can be subdivided into two main portions: visceral (involving internal organs and abdominal region) and subcutaneous, a fat layer located just below the skin. These portions are differently distributed throughout the body according to sex (3).

Sexual dimorphisms favor reproduction and the perpetuation of the species (4,5). It can also explain why males and females may have distinct susceptibility to different types of diseases and be affected by different patterns of their manifestation and progression. There are thousands of sexually dimorphic genes in countless tissues. Over 16,600 of them are found in the adipose tissue, and over 11,000 are differentially expressed according to sex (6,7). The differences also involve redox homeostasis in different physiological conditions (8). Reactive oxygen species (ROS) are produced by the activities of mitochondria and NADPH oxidases (NOX), among other sources, and must be proportionally neutralized by antioxidant systems, including the antioxidant enzymes catalase (CAT), superoxide dismutase (SOD), and glutathione peroxidase (GPx) (9,10).

Only a few studies to date have focused on the redox balance in different depots, such as visceral and subcutaneous fat pads in males and females. Therefore, aiming to broaden the understanding of how differently some redox homeostasis parameters manifest in male and female rats, our study investigated the activity and expression of NADPH oxidases and antioxidant enzymes in the retroperitoneal and subcutaneous fat pads in control rats.

## MATERIALS AND METHODS

### Animals

A total of 12 Wistar rats of both sexes (6 males and 6 females), aged 4 months and weighing 200-300 g, were analyzed. The animals were kept in separate boxes with controlled temperature (22-25 ºC), 12-hour light/12-hour dark cycles, and water and food *ad libitum*, undergoing no experimental intervention during the follow-up period. They were euthanized in their 16th week of life after 8 hours of fasting. The retroperitoneal (RP) and subcutaneous (SC) fat pads were collected, weighed, and stored in a −80 ºC freezer for further analysis.

The research was carried out with approval by the Ethics Committee for the Use of Animals from the State University of Ceará (number 2636736/2017).

### Total reduced thiol

The samples were homogenized in 5 mM Tris-HCl buffer (pH 7.4) containing 0.9% NaCl (w/v) and 1 mM EDTA, followed by centrifugation at 720 g for 10 minutes at 4 ºC. The same amount of tissue was used for each group and the infranatant aliquots were collected.

Total reduced thiol groups were determined using a spectrophotometer (Hitachi U-3300) and 5,5’-dithiobis(2-nitrobenzoic acid) (DTNB). Briefly, thiols react with DTNB, cleaving the disulfide bond to yield 2-nitro-5-thiobenzoate (NTB-), which ionizes to NTB^2-^ in water at neutral and alkaline pH. The NTB^2-^ was quantified using a spectrophotometer at 412 nm (11).

### Superoxide dismutase activity

The total activity of SOD was determined by the cytochrome C reduction method using the xanthine-xanthine oxidase system, producing an increase in light absorption at 550 nm. The sample containing SOD converts superoxide into H_2_O_2_, thus preventing the reduction of cytochrome C. A 50% decrease in the cytochrome C reduction rate represents one SOD unit, which is expressed in units per milligram of protein (U.mg^−1^) (12).

### Catalase activity

The CAT activity was assayed by measuring the disappearance of H_2_O_2_, which forms water and oxygen, causing a decrease in light absorption at 240 nm (13). After a 1-minute reading, CAT activity was measured as the difference between the decrease in absorbance rate with and without the sample, reflecting the amount of consumed H_2_O_2_, according to its molar extinction coefficient (43.6 M^−1^). The CAT activity was expressed in units per milligram of protein (U.mg-1), in which one CAT unit represents 1 micromole of H_2_O_2_ consumed per minute.

### Glutathione peroxidase activity

The GPx activity was determined by measuring cytochrome C reduction at 550 nm. Briefly, this method is based on hydroperoxide conversion to water by GPx, when the reduced (free) glutathione (GSH) is oxidized to form glutathione (GSSG) at the expense of NADPH oxidation. The decrease in light absorption at 340 nm promoted by NADPH oxidation was evaluated for 5 minutes. The GPx activity was expressed in units of micromole of oxidized NADPH per minute.

### Microsomal NADPH oxidase activity

The NOX activity was quantified in the microsomal fraction (MF) using the Amplex Red/horseradish peroxidase assay (Molecular Probes, Invitrogen). Isolation of MF was carried out according to a previously validated method that allows the isolation of all the intracellular content (14). Since NOX complexes bind to cell membranes, MF is an important measure of their activity, as it comprises organelles and intracellular vesicles, along with their membranes.

The fat pads were homogenized in buffer (pH 7.2, containing 0.25 M sucrose, 0.1 dithiothreitol, 1 mM EGTA, 50 mM sodium phosphate, 5 mg/mL aprotinin, and 34.8 mg/mL phenylmethanesulfonyl fluoride [PMSF]) and centrifuged at 12,000 g for 15 minutes at 4 °C. Then, the supernatant was centrifuged twice at 100,000 g for 35 minutes at 4 oC to yield the MF, which was resuspended in 50 mM sodium phosphate buffer (pH 7.2, containing 0.25 M sucrose, 2 mM MgCl_2_, 5 mg/mL aprotinin, and 34.8 mg/mL PMSF) and stored at −80 °C. In order to evaluate NOX activity, MF was incubated in 150 mM sodium phosphate buffer (pH 7.4) containing 100 U/mL SOD (Sigma, USA), 0.5 U/mL horseradish peroxidase (Roche, Indianapolis, IN, USA), 50 µM Amplex Red (Molecular Probes, Eugene, OR, USA), 1 mM EGTA, and 1 mM NADPH. Fluorescence was measured in a microplate reader (Victor X4; PerkinElmer, Norwalk, CT, USA) at 30 °C, using excitation at 530 nm and emission at 595 nm (15). The enzymatic activity was expressed as nanomoles of H_2_O_2_ per hour per milligram of protein (nmol.h^−1^.mg^−1^). Protein concentration was determined using the Bradford assay (14).

### Real-time PCR

Following the previous steps, RNA was extracted from samples using the Direct-zol RNA Miniprep Plus kit (Zymo Research, CA, USA) according to the manufacturer's recommendations. To obtain cDNA, the extracted RNA samples (1 µg) were pretreated with DNAse. Subsequently, 10 µL of a mixture containing 4 μL of buffer, 1.2 μL of MgCl_2_, 1.0 μL of dNTP, 0.5 μL of RNAsin, 1.0 μL of GoScript, and 2.3 μL of H_2_O was added. The mixture was incubated in a thermocycler with specific temperature and time and, immediately afterward, the cDNA was transferred to a −20 ºC freezer. Quantitative analysis of mRNA levels was performed using a quantitative PCR (qPCR) technique with a C1000 Touch thermal cycler (Bio-Rad Laboratories, CA, USA). The assays were performed in duplicates, using 3 µL of cDNA (obtained during reverse transcriptase) and 9 µL of a mixture containing 6 µL of Syber Green (Life Technologies, CA, USA), 0.25 µL each of reverse and forward primers (Table 1) with a final concentration of 200 nM, and 2.5 µL of H_2_O, resulting in a final reaction volume of 12 µL.

**Table 1:** List of reverse and forward primers used in the real-time polymerase chain reaction (PCR) assay

	Reverse	Forward
*Actb*	TCAGGTCATCACTATCGGAATG	TTTCATGGATGCCACAGGATTC
*Gus*	TGTCTGCGTCATATCTGGTATTG	GGTCGTGATGTGGTCCTGTC
*Sod1*	CTTCCAGCATTTCCAGTCTTTG	TGTGTCCATTGAAGATCGTGTG
*Sod2*	CAAAAGACCCAAAGTCACGC	GGACAAACCTGAGCCCTAAG
*Gpx1*	GAAGGTAAAGAGCGGGTGAG	AATCAGTTCGGACATCAGGAG
*Cat*	TTGAAAAGATCTCGGAGGCC	CAAGCTGGTTAATGCGAATGG
*Nox1*	ATTCGTCCATCTCTTGTTCCAG	AAGTGGCTGTACTGG
*Nox2*	CGAGTCACAGCCACATACAG	CAATTCACACCATTGCACATC
*Nox4*	GGTTTCCAGTCATCCAGTAGAG	TCCATCAAGCCAAGATTCTGAG

Abbreviations: *Actb*, actin beta (UGO Gene Nomenclature Committee abbreviation); *Cat*, catalase; *Nox1*, NADPH oxidase 1; *Nox2*, NADPH oxidase 2; *Nox4*, NADPH oxidase 4; *Gpx1*, glutathione peroxidase; *Gus*, β-glucuronidase reporter gene system; *Sod1*, superoxide dismutase 1; *Sod1*, superoxide dismutase 2.

### Statistical analysis

The results are expressed as mean ± standard error of the mean and were analyzed by two-way analysis of variance (ANOVA) followed by Bonferroni's multiple comparison test (except for body weight data, for which unpaired Student's *t* test was used).

Statistical analyses were carried out using GraphPad Prism (version 7.0, GraphPad Software Inc., San Diego, CA, USA) and the minimum level of significance was set at p < 0.05. All statistical analyses and p values are presented in the Supplementary Table.

## RESULTS

### Body weight and fat pads’ weight

At 16 weeks of life, body weight was greater in male rats compared with female rats. The absolute weights of the fat pads were also greater in males compared with females. When the weights of the fat pads were normalized by body weight, RP and SC fat pads of males were reduced compared with those of females (Table 2).

**Table 2:** Body weight and adiposity of the female (n = 6) and male (n = 6) rats analyzed in the study*

Variables		Male rats	Female rats
Body weight (g)		317.2 ± 11.71	211.2 ± 6.14***
RP fat pad	Weight (g)	0.6434 ± 0.038	0.6578 ± 0.059**
	Relative (%)	0.204 ± 0.012	0.313 ± 0.029**
SC fat pad	Weight (g)	0.8929 ± 0.059	0.7852 ± 0.07**
	Relative (%)	0.282 ± 0.017	0.373 ± 0.034**

* Total body weight at 16 weeks of life in male and female animals; absolute weights of subcutaneous (SC) and retroperitoneal (RP) fat pads in male and female animals aged 16 weeks; and relative weight measurements, normalized by body weight, in the SC and RP. The data are expressed as mean ± standard error of the mean.

** Difference between sexes (p < 0.05).

*** Difference between sexes (p < 0.0001). Unpaired Student's *t* test (for body weight). Repeated-measured two-way analysis of variance (ANOVA) followed by Bonferroni's multiple comparison test.

### Activity and mRNA levels of NADPH oxidases in the microsomal fraction

**Figure 1:**
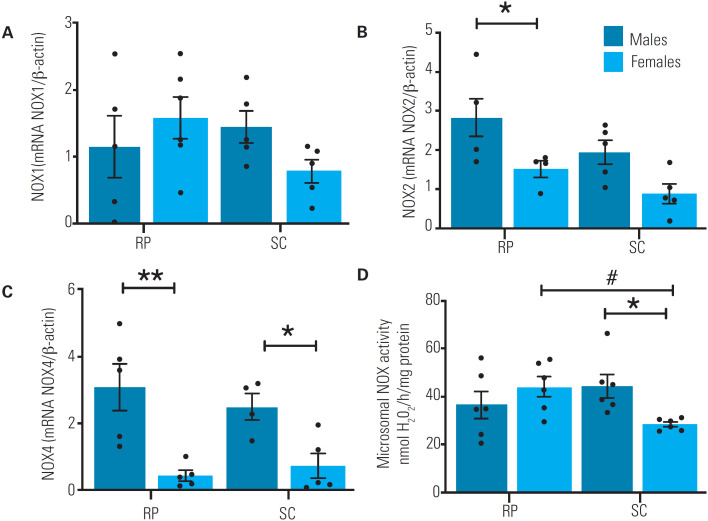
Levels of NADPH oxidase mRNA in subcutaneous (SC) and retroperitoneal (RP) fat pads of male (n = 6) and female (n = 6) rats aged 16 weeks. (**A**) Expression levels of NADPH 1 (NOX1) mRNA. **(B**) Expression levels of NADPH 2 (NOX2) mRNA. (**C**) Expression levels of NADPH 4 (NOX4) mRNA. All PCR data were assessed using real-time PCR and normalized to β-actin mRNA expression. (**D**) Microsomal NOX activity. The H_2_O_2_ production was determined in the microsomal enriched fraction using the Amplex Red/horseradish peroxidase assay. *Sex differences in comparisons within the same tissue (p < 0.05) **Sex differences in comparisons within the same tissue (p < 0.001). #Tissue differences in comparisons within the same sex (p < 0.05). Repeated-measures two-way analysis of variance (ANOVA) followed by Bonferroni's multiple comparison test. The data are expressed as mean ± standard error of the mean.

No difference was observed between males and females regarding the mRNA expression of *Nox1* (Figure 1A). In contrast, the mRNA expression of *Nox2* in the RP fat pad (Figure 1B) and the mRNA expression of *Nox4* in both SC and RP fat pads (Figure 1C) were higher in males than females. The production of H_2_O_2_ in the MF of the SC fat pad was higher in males than females (Figure 1D). Only female rats exhibited higher H_2_O_2_ production in the MF of the RP fat pad compared with the SC fat pad (Figure 1D).

### Levels of mRNA of antioxidant enzymes

**Figure 2:**
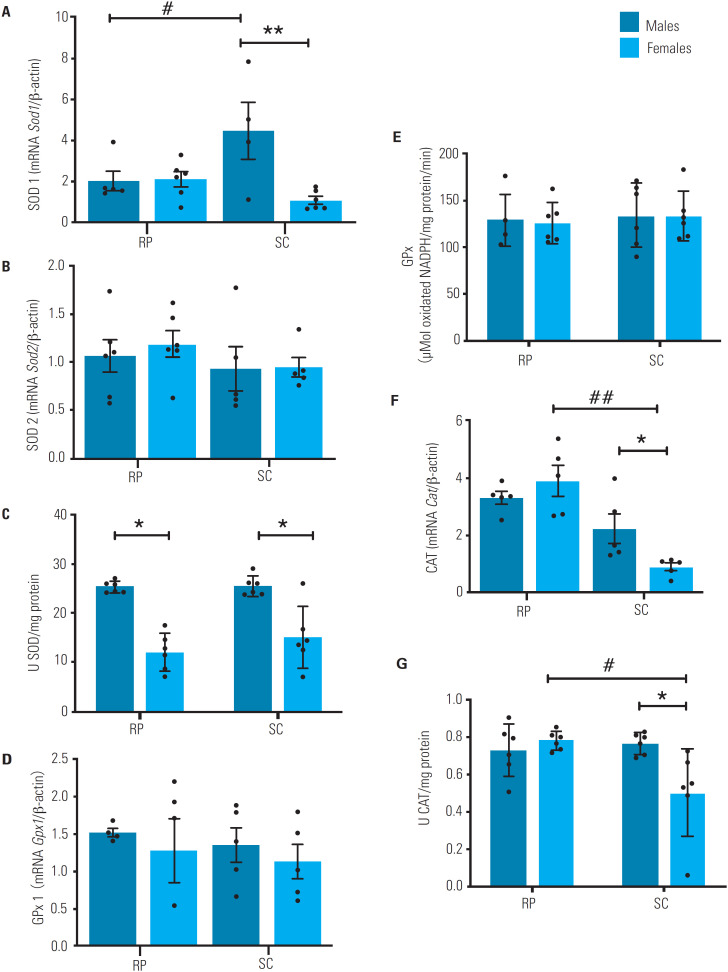
Activity and mRNA levels of antioxidant enzymes in subcutaneous (SC) and retroperitoneal (RP) fat pads of male (n = 6) and female (n = 6) rats aged 16 weeks. (**A**) Superoxide dismutase 1 (*Sod1*) mRNA levels. (**B**) Superoxide dismutase 2 (*Sod2*) mRNA levels. (**C**) Superoxide dismutase (SOD). (**D**) Glutathione peroxidase 1 (*Gpx1*) mRNA level. (**E**) Glutathione peroxidase (GPx) activity levels. (**F**) Catalase (*Cat*) mRNA levels. (**G**) CAT activity. The mRNA data were assessed using real-time PCR and normalized to β-actin mRNA expression and expressed as mean ± standard error of the mean. The activity of antioxidant enzymes was measured using spectrophotometry, and the results were expressed as mean ± standard error of the mean. *Sex differences in comparisons within the same tissue (p < 0.05). **Sex differences in comparisons within the same tissue (p < 0.001). #Tissue differences in comparisons within the same sex (p < 0.05). ##Tissue differences in comparisons within the same sex (p < 0.0001). Repeated-measures two-way analysis of variance (ANOVA) followed by Bonferroni's multiple comparison test.

The mRNA expression of *Sod1* in the SC fad pad was higher in males than in females (Figure 2A), but no difference between sexes was observed in the expression of *Sod2* and *Gpx1* (Figures 2B and 2D). Additionally, *Cat* in the SC fad pad had higher mRNA expression in males than females (Figure 2F). Only female rats exhibited higher mRNA levels of *Cat* in the RP fat pad compared with the SC fat pad (Figure 2F).

### Activity of antioxidant enzymes

Both SC and RP fat pads had higher total SOD activity in males than females (Figure 2C). No significant difference between sexes was observed regarding GPx activity in the SC or RP (Figure 2D). Sex differences in total CAT activity were only present in the SC fat pad, in which the activity was higher in males than females (Figure 2G). Within each sex group, only females exhibited different CAT activity levels between fat pads, as they were higher in the RP than in the SC fat pad (Figure 2G).

### Total reduced thiol

**Figure 3:**
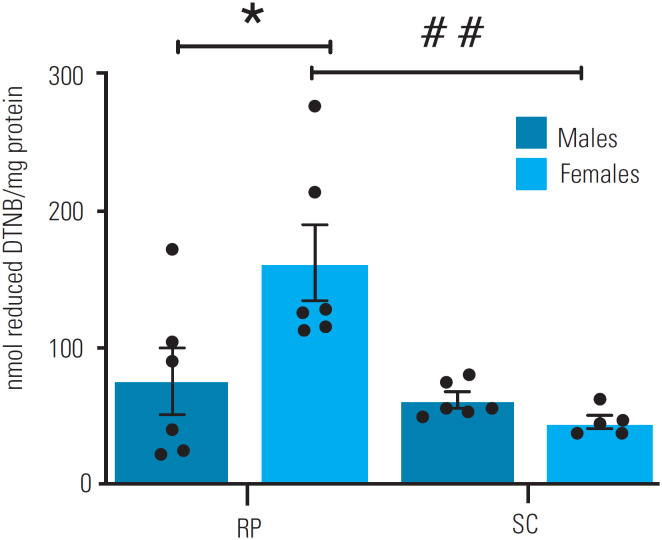
Total reduced thiol in subcutaneous (SC) and retroperitoneal (RP) fat pads of male (n = 6) and female (n = 6) rats aged 16 weeks. Reactive protein thiol levels in SC and RP fat pads were assessed by measuring total sulfhydryl groups from a reaction of thiols with DTNB spectrophotometry. The data are expressed as mean ± standard error of the mean. *Sex differences in comparisons within the same tissue (p < 0.05). ##Tissue differences in comparisons within the same sex (p < 0.0001). Repeated-measures two-way analysis of variance (ANOVA) followed by Bonferroni's multiple comparison test.

Females had higher levels of reduced thiol in the RP fat pad compared with males (Figure 3). When thiol quantification was compared between RP *versus* SC fat pads within each sex, reduced thiol levels were higher in the RP compared with the SC fat pad in females, whereas no difference was observed in males.

## DISCUSSION

Our study demonstrated greater body weight gain but lower relative adiposity among young adult male *versus* female rats. This finding corroborates the emerging understanding of sex dimorphism in adiposity, explained by complex factors that include mainly sex hormones and the endocrine regulation of energy metabolism (16-18). For example, estrogen is proven to affect the distribution of adipose tissue, regulating the expression of lipogenic genes, while androgens are classically associated with decreased total and central adiposity by mechanisms that involve both increasing lipolysis and inhibiting lipoprotein lipase activity (19,20). The resting metabolic rate differences between males and females must also be taken into account, as differences in body composition itself may influence total resting energy expenditure (21,22).

Our research group has previously confirmed the finding of sex differences regarding changes in redox parameters triggered by diet-induced obesity (23). In our previous study, MFs of subcutaneous WAT from male and female rats exposed to a standard diet showed no differences in NOX activity or *Nox4* mRNA expression (23), in contrast to the present study, which showed decreased NOX activity and *Nox4* mRNA expression in subcutaneous WAT of female rats. The conflicting results between our previous study (which included 8-month-old animals) and the present study (which included 4-month-old animals) may be related to obesity in the animals in the first study and explained by a tendency of control animals to gain weight and become obese over time. With a focus on non-obese animals in the present study, we observed a sex difference in *Nox4* mRNA expression, which was *higher* in both RP and SC fat pads of male rats than female rats. Additionally, male rats, compared with female rats, showed higher *Nox2* expression in the RP fat pad and increased NOX activity in the MF of the SC fat pad. Notably, greater H_2_O_2_ production and NOX activity have been previously shown in the WAT of male rats (23).

Of note, NOX comprises a family of transmembrane enzymes responsible for ROS production in most tissues, and NOX4 is the main NOX isoform in adipose tissue for H_2_O_2_ production (24). The activity of these enzymes mediates diverse adaptations and responses that require selective communication across the cell membrane and has a crucial role in configuring the redox status. (24,25). Their mechanisms are known to include precisely the production of O2^•-^ by endothelial subunits of enzymatic structures that generate reduced NADPH. Catalyzed by SOD, O2^•-^ can undergo dismutation into H_2_O_2_, a molecularly more stable ROS with a relatively longer half-life that has a high affinity for other molecules, cell structures and, upon receiving an additional electron, generating the anion superoxide (OH^•^), one of the most damaging ROS (25). Since we evaluated NOX activity in isolated MF, it is possible that the intracellular production of ROS by NOX appeared to be sex-specific but had a different type of mechanism that is still unclear. This sex dimorphism has not been explained specifically in the adipose tissue, but likely involves the regulation by sex hormones, probably due to a previously described negative correlation of β-estradiol with H_2_O_2_ levels downregulating some NOX subunits and inhibiting NOX activity (18,26,27). Also, androgens have been described as enhancers of ROS production and oxidative stress in tissues such as cardiomyocytes via androgen receptors and both nongenomic and genomic mechanisms (16). Whether these effects explain the observed phenomenon in WAT remains to be elucidated.

Important explanation for the differences in redox parameters also involves the enzymatic antioxidant system, as it promotes interactions for the establishment of a redox state. In the present study, we assessed the redox status by quantifying total thiols and then investigated the response to a possible oxidative stressor scenario. The results demonstrated sexual dimorphism in mRNA expression of SOD and CAT in the SC fat pad. Male rats had greater SOD and CAT activities in the SC fat pad and greater SOD activity in the RP compared with female rats. These sex differences occurred together, with no between-group differences in thiol levels, suggesting that males had a relatively greater increase in antioxidant activity in order to maintain their redox state. The increased ROS availability in males may result from specific activation of redox-sensitive transcription factors, such as nuclear factor (erythroid-derived 2)-like 2 (NRF2), activator protein-1 (AP-1), and nuclear factor kappa B (NF-kB), which, in turn, can regulate the expression of genes encoding antioxidant enzymes (27,28). A greater NOX production of H_2_O_2_, as found in our study, could be a possible disturber of the redox state because, when it occurs in the local context, redox interactions occur to neutralize the oxidative potential of ROS. This would explain the upregulated antioxidant defense. Further investigations concerning differences in how this redox relationship may or may not cause specific oxidative damages are necessary to broaden this understanding.

As mentioned earlier, the sex differences in redox parameters could involve endocrine regulation via sex hormones, since β-estradiol is proven to act as a redox-protection factor, whereas androgens seem to promote a pro-oxidative effect (8,17,28,29). This effect occurs through well-known mechanisms that often involve the nuclear transcription of *Sod2* by activating the MAPK and NFkB pathways (30-33), consequently affecting the intrinsic pathways of redox regulation. Indeed, our results demonstrated sex differences in SOD gene expression and activity. These properties could also be related to the increased NOX production of ROS in males. However, such correlation needs to be investigated specifically in the adipose tissue for a clear elucidation of their mechanisms.

It is important to mention that the establishment of oxidative stress is constantly associated with the unregulated secretion of cytokines that comes with cell damage (10,25,33,34). Since byproducts of lipid peroxidation and protein carbonylation can damage some crucial intracellular structures of the adipose tissue, compromising it functionally, secretion of cytokines involved in inflammatory signaling further stimulates ROS production. Cytokines can trigger NOX activity, sending positive feedback to the entire oxidative stress cycle (25,33,34). Future studies should evaluate potential sexual dimorphisms in these relationships.

Our study further compared within-group SC *versus* RP, and the results suggested occasional tissue heterogeneity. The RP of females had an overall lower expression of NOX genes, but still, H_2_O_2_ production by the RP was similar in both sexes, while the male *versus* female differences in NOX expression in the SC corresponded with the H_2_O_2_ production. Expression of *Sod1* differed between both fat pads in males, which had higher levels in the SC compared with the RP. Additionally, *Cat* expression was considerably higher in the RP compared with the SC in females, while thiol was higher in the RP than the SC in males. A well-known heterogeneity regarding metabolic and redox parameters exists between adipose fat pads in general, and the observed differences could also involve sex-related distinct mechanisms by endocrine regulation of energy metabolism and redox interactions, although more specific mechanisms still need to be elucidated.

In conclusion, our study investigated sex differences in specific redox parameters between male and female rats and found that male WAT had a predominant pro-oxidative redox state, with greater NOX-dependent ROS production, but with a proportional antioxidant enzymatic response. Specifically, the RP of males compared with that of females had greater *Nox2* and *Nox4* expression, along with greater SOD activity, and their SC had greater *Nox4* expression and higher intracellular H_2_O_2_ production together with greater expression and activity of both SOD and CAT. In addition to the well-known heterogeneity of the adipose tissue and its metabolic patterns, some redox parameters of WAT seem to occur in a sex-specific manner. These findings are important evidence for the ongoing elucidation of specific mechanisms and correlation between sex differences and the genesis of some metabolic pathologies, as well as the susceptibility of each sex in developing them.

## Supplementary Table Results of statistical analyses

**Table:**

	Sex	Fat Pad				
		Retroperitoneal		Subcutaneous		p value
Nox1 (mRNA NOX1/β-actin)	Male	1.15	± 1.03	1.45	± 0.53	0.7001
	Female	1.58	± 0.76	0.78	± 0.39	0.0045*
	p value	>0.9999		0.8228		
Nox2 (mRNA NOX2/β-actin)	Male	2.83	± 1.08	1.94	± 0.68	0.9483
	Female	1.51	± 0.42	0.88	± 0.54	0.0203*
	p value	0.0485*		0.1386		
Nox4 (mRNA NOX4/β-actin)	Male	3.09	± 1.57	2.5	± 0.78	>0.9999
	Female	0.45	± 0.35	0.73	± 0.83	0.3962
	p value	0.0012*		0.0308*		
Microsomal NOX activity nmol H_2_O_2_/h/mg protein	Male	36.61	± 13.76	44.44	± 11.83	0.2524
	Female	44.19	± 10.35	28.43	± 2.46	0.5383
	p value	0.4014		0.0314*		
Sod1 (mRNA SOD1/β-actin)	Male	2.03	± 1.06	4.49	± 2.79	0.7715
	Female	2.10	± 0.90	1.08	± 0.47	0.9519
	p value	0.9998		0.0075*		
Sod2 (mRNA SOD2/β-actin)	Male	0.94	± 0.23	0.93	± 0.51	0.379
	Female	1.19	± 0.34	1.07	± 0.41	0.0343*
	p value	0.7268		0.9356		
U SOD/mg protein	Male	25.4	± 0.49	25.63	± 0.83	0.0075*
	Female	12.12	± 1.57	15.14	± 2.57	0.6474
	p value	<0.0001*		0.0007*		
Gpx1 (mRNA GPx1/β-actin)	Male	1.52	± 0.12	1.35	± 0.51	0.9999
	Female	1.28	± 0.85	1.08	± 0.47	0.9439
	p value	0.9156		0.8808		
GPx (μmol oxidated NADPH/mg protein/min)	Male	128.78	± 11.41	134.4	± 14.05	0.9996
	Female	126.19	± 8.99	133.53	± 0.11	0.5416
	p value	0.9985		>0.9999		
Cat (mRNA CAT/β-actin)	Male	3.32	± 0.49	2.24	± 1.15	0.9734
	Female	3.92		± 1.19		0.9421
	p value	0.4646	0.0408*			0.89
U CAT/mg protein	Male	0.73	± 0.06	0.77	± 0.02	0.9854
	Female	0.78		± 0.02		0.9686
	p value	0.929	0.0213*			0.5
nmol reduced DTNB/mg protein	Male	162.2	± 67.98	45.64	± 10.34	0.2496
	Female	75.56	± 58.55	61.34	± 12.91	<0.0001
	p value	0.0360*		>0.9999		

The values are expressed as mean ± standard error of the mean (SEM).

* Analysis of statistical significance – measured using two-way analysis of variance (ANOVA) followed by Bonferroni's multiple comparison test – between sexes (male versus female) and between tissues (retroperitoneal versus subcutaneous) of each sex.
